# Editorial: Zoonoses - a one health approach

**DOI:** 10.3389/fpubh.2023.1332600

**Published:** 2023-12-04

**Authors:** Balbir B. Singh, Ranjani Somayaji, Rajnish Sharma, Herman W. Barkema, Baljit Singh

**Affiliations:** ^1^Centre for One Health, Guru Angad Dev Veterinary and Animal Sciences University, Ludhiana, Punjab, India; ^2^Department of Community Health Sciences, Cumming School of Medicine, University of Calgary, Calgary, AB, Canada; ^3^One Health at UCalgary, University of Calgary, Calgary, AB, Canada; ^4^Department of Veterinary Biomedical Sciences, University of Saskatchewan, Saskatoon, SK, Canada

**Keywords:** zoonoses, antimicrobial resistance (AMR), one health, emerging zoonoses, endemic zoonoses

Zoonoses are defined as “those diseases and infections [*the agents of* ] which are naturally transmitted between [*other*] vertebrate animals and man” (1). Zoonotic diseases are responsible for considerable negative health impacts and economic consequences (2). Many recently discovered emerging pathogens, such as SARS-CoV-2, Ebola, and Nipah virus, are zoonotic in nature (3), and the cross-species pathogen spillover from non-human animal hosts to humans could be associated with the majority of human infectious diseases and pandemics (4–6). An increase in human–animal interactions, contacts, and the human–animal interface may increase the risk of zoonotic pathogens in manifold human populations (7). Furthermore, societal characteristics, ecological disturbances, environmental stress, and climate change have strong association with zoonoses (8–11). In contrast to the diseases caused by single-host pathogens, prevention, control, and eradication of the diseases caused by zoonotic pathogens (which involve two or more hosts) are often difficult due to the complex pathogen transmission matrix and the real probability of the presence of multiple reservoir hosts. Interventions at the critical control points (CCPs) of zoonotic diseases are essential and need multi-sectoral engagement. Formal or informal slaughterhouses, wet markets, and carcass disposal sites are important CCPs that need attention at the animal–environment interface. A lack of coordination among environmental, animal, and public health sectors, nationally and internationally, has the potential to seriously undermine zoonotic disease control programs. In addition, antimicrobial resistance (AMR) has complicated the prevention and control of zoonoses due to the emerging general consensus to restrict the use of antimicrobials in food and companion animals (3). The One Health approach takes a systems view of these complex infectious disease problems, recognizing interconnections of people and animals in their shared environments. The One Health lens encompasses the social, economic, cultural, physical, built, and political environments that can either promote or inhibit the prevention and control of these diseases. A One Health approach can identify and implement robust and meaningful solutions to improve the health and wellness of people, animals, and the environment within existing social, economic, and political contexts (12).

The current issue “*Zoonoses - a one health approach*” of “*Frontiers in Public Health*” focuses on the importance of the One Health approach in tackling complex problems such as AMR and zoonosis. The topics covered include an opinion article (Singh et al.) on the historic developments associated with the standard definition of *zoonosis*, discrepancies in the usage of the term *zoonosis*, and suggestions for the introduction of additional terms such as *Olazoonosis, Akrizoonosis, Anakrizoonosis, Zoizoonosis, Nekrózoonosis*, and *Pidózoonosis* in the published literature. The importance of One Health approaches for zoonotic disease surveillance is highlighted by Riley et al. using zoonotic disease notifications from the period of 1996–2021 in Aboriginal and Torres Strait Islander populations in Australia.

Emerging and re-emerging viral diseases are a serious threat in Southeast Asia, and there is a need to understand the drivers of disease emergence and transmission to human and animal populations. In this issue, the application of One Health to meet this challenge in Southeast Asia has been elaborately discussed (Saba Villarroel et al.). Wet markets have proven to be a critical source of disease emergence, and Islam et al. in this issue have discussed the estimated risk factors associated with avian influenza virus (H5 and H9) contamination in live bird markets located in rural and peri-urban regions in Bangladesh.

The One Health approach is also crucial for the control and elimination of neglected tropical diseases (13). The utility of a One Health model in the detection of canine rabies cases when coupled with an integrated bite case management program in Vietnam (Ross et al.) is thus showcased in this issue. However, several issues might arise in One Health-influenced collaborations. In this issue, Suschinel et al. present the challenges in conducting an international research project on leishmaniasis in Colombia, including collaborations amongst public health institutions and dog owners.

Endemic zoonoses are responsible for a substantial burden on human and animal health, particularly in developing countries, and are deterrent to the economy (14). Preventive One Health interventions have been recommended in controlling endemic zoonoses (15). In this issue, Ayalew et al. have presented a situation assessment of zoonotic tuberculosis in Ethiopia. It is recommended to advocate for a One Health approach for the control of endemic zoonoses as the lack of knowledge on zoonoses such as brucellosis in medical professionals could result in disease misdiagnosis (Qin et al.).

Similar to zoonoses, AMR is an important One Health priority because it is a global threat to the One Health ecosystem (16). Therefore, a longitudinal One Health analysis of antimicrobial use and resistance patterns in humans and food-producing animals residing in Europe has been presented (Rahman and Hollis). The epidemiologic aspects of AMR in companion animals in the United States to inform One Health AMR programs have also been included (Sobkowich et al.). Lastly, the application of a One Health approach to understand the perceptions of different stakeholders on antimicrobial stewardship has been undertaken by identifying the associated drivers and barriers in Canada (McCubbin et al.).

In summary, the Research Topic “*Zoonoses - a one health approach*” highlights many important aspects of the One Health approach to control AMR and zoonosis.

## Author contributions

BBS: Conceptualization, Writing—original draft. RSo: Supervision, Writing—review & editing. RSh: Writing—review & editing. HB: Supervision, Writing—review & editing. BS: Supervision, Writing—review & editing.
